# School-Based Caries Prevention Programs and Recruitment of High-Risk Pediatric Medicaid Populations

**DOI:** 10.1001/jamanetworkopen.2026.5996

**Published:** 2026-04-09

**Authors:** Shulamite S. Huang, Ryan R. Ruff, Heather T. Gold, Scarlett Sijia Wang

**Affiliations:** 1 Department of Epidemiology and Health Promotion, New York University College of Dentistry, New York, New York; 2 Division of Community Oral Health, University of Pennsylvania School of Dental Medicine, Philadelphia; 3 Section on Value and Effectiveness Research, Department of Population Health, New York University Grossman School of Medicine, New York, New York; 4 New York University, Wagner School of Public Service, New York, New York

## Abstract

**Importance:**

Pediatric dental-related emergency department visits have drastically increased in recent years. School-based caries prevention programs (SCPPs) aim to address unmet dental needs, yet it is unclear whether they effectively reach high-risk populations.

**Objective:**

To determine whether children with prior dental care utilization are more likely than children without prior utilization to participate in SCPPs.

**Design, Setting, and Participants:**

This cross-sectional study, conducted between June 2023 and October 2025, linked 2019 SCPP clinical trial data and 2016 to 2019 Medicaid claims data to examine disparities in sociodemographic characteristics and dental and dental-related medical care utilization prior to SCPP implementation between participants and nonparticipants. SCPPs were implemented in 47 primary schools in New York, New York, primarily in the Bronx, which was deemed to have the highest risk of tooth decay in the city. Participants included children aged 5 to 13 years in 2019 residing in the Bronx and continuously enrolled in Medicaid between 2018 and 2019.

**Exposures:**

Dental and dental-related medical care utilization in 2018, the year prior to SCPP implementation, demographic factors, individual-level clinical dental needs, and health system factors.

**Main Outcomes and Measures:**

The primary outcome was SCPP participation in 2019. Adjusted logistic regression was used to assess whether prior dental care use was independently associated with participation.

**Results:**

The study included 63 217 children in total (62 187 nonparticipants and 1030 participants), with a mean age of 7.7 years (95% CI, 7.6-7.7 years). There were 30 590 female children (48.4%), 1852 Asian children (2.9%), 13 926 Black children (22.0%), 31 620 Hispanic children (50.0%), and 1988 White children (3.2%). In multivariable analysis, compared with any dental visits, having no dental visits prior to SCPP implementation was associated with 17% lower odds of participating (adjusted odds ratio, 0.83; 95% CI, 0.71-0.96). Compared with having any dental emergencies, having no dental emergencies was associated with 32% increased odds of participating (adjusted odds ratio, 1.32; 95% CI, 1.08-1.62). Mitigating selection from a statewide SCPP rollout was estimated to save up to $2.4 million in nontraumatic dental-related emergency department visits.

**Conclusions and Relevance:**

In this cross-sectional study of NY Medicaid claims data linked to SCPP participation data, there was evidence that children at high risk of tooth decay who are most in need of dental preventive services have a lower likelihood of participating in school-based prevention programs. More evidence is needed to identify successful strategies for recruitment of high-need children.

## Key Points

**Question:**

Are children with prior dental care utilization more likely than those without prior utilization to participate in school-based caries prevention programs (SCPPs)?

**Findings:**

In this cross-sectional study of 63 217 children, having no prior dental visits was associated with a statistically significant 17% lower odds of SCPP participation compared with any prior visits. Having no emergency dental utilization was associated with a statistically significant 32% increased odds of SCPP participation compared with any emergency dental utilization.

**Meaning:**

These findings suggest that children with high risk of unmet dental needs have a lower likelihood of participating in SCPPs; however, more evidence is needed to optimize strategies to recruit high-need children.

## Teaser

This cross-sectional study examines whether children with prior dental care utilization are more likely than children without prior utilization to participate in school-based caries prevention programs.

## Introduction

Dental caries (tooth decay) remains the most prevalent disease among children, affecting more than 20% of children aged 2 to 5 years.^1^ Although there has been a steady decline in untreated decay over the past 50 years, this trend has recently plateaued.^2^ Additionally, in recent years, emergency department (ED) admissions for nontraumatic dental conditions increased among children younger than 14 years, from 27.3 to 43.1 admissions per 10 000 children between 2019 and 2022.^3,4^ Because EDs are generally limited to providing palliative care and pain relief, requiring external referrals to dental offices for definitive treatment, these ED visits represent a drain on health care resources. With the decline of pediatric capacity at general hospitals,^5,6,7^ pediatric ED readiness,^8^ and Medicaid funding, identifying levers to decrease unmet dental needs is critical to avoid further burdening EDs.

School-based caries prevention programs (SCPPs) are a primary strategy to increase access to dental care in pediatric populations. Programs providing primary and/or secondary preventive treatment, such as dental sealants, silver diamine fluoride, and atraumatic restorations, have been found to reduce the risk of caries by 30% and are cost-effective.^9,10,11,12,13^ They may also prevent children from seeking care in EDs. However, providing treatment and/or conducting research on SCPPs in schools requires parental informed consent, which may generate selection bias issues in evaluating SCPPs, as research-averse and dental-averse children and families may also be those more likely to be high risk.^14^ Hence, it is unclear whether SCPPs reach children who are most in need, and research on participation rates in children with high caries risk is limited.^15^ If programs are more likely to attract those with low oral health risk, then current estimates of the clinical effectiveness and cost-effectiveness of SCPPs may be biased.

Using data from 2 large SCPPs and a pragmatic clinical trial linked to Medicaid claims data, we assessed whether program participants were more likely to have had prior year dental care utilization compared with the general Medicaid population. The selected SCPPs provided preventive and therapeutic dental care to targeted populations at high caries risk. We hypothesized that children with any experience of using dental care in the year before SCPP implementation would be more likely to participate in SCPPs, given their increased familiarity with dental procedures. We further tested for any differences in sociodemographic characteristics and disparities in dental care utilization before SCPP implementation between participants and nonparticipants and estimated the impact of removing SCPP selection bias on statewide dental ED visit changes.

## Methods

This cross-sectional study was granted an exemption by the New York University School of Medicine institutional review board. The study follows the Strengthening the Reporting of Observational Studies in Epidemiology (STROBE) reporting guideline.

### SCPP Setting

SCPPs providing primary and/or secondary prevention in schools can vary widely in (1) the treatments delivered (ie, fluoride varnish, sealants, silver diamine fluoride, and/or atraumatic restorations), (2) staffing arrangements due to variation in state scope of practice laws, and (3) frequency of visits. In this study, we examine 2 different SCPP designs that were implemented through the CariedAway research study, a cluster-randomized noninferiority trial. CariedAway was implemented in 47 primary schools (prekindergarten through eighth grade) in New York, New York (hereafter, NYC), primarily in the Bronx, deemed to be at the highest risk of caries in NYC (>80% students participating in free or reduced lunch and primarily Hispanic or Latino populations).^16^ Caries prevention in the 2 CariedAway programs was implemented on February 1, 2019, disrupted early March 2020 because of the COVID-19 pandemic, and later restarted and continued through the first half of 2023. All students enrolled in a school participating in the CariedAway study were eligible to receive care.^13^ Schools were block randomized to deliver 1 of 2 caries prevention protocols twice a year, either (1) the application of a 38% concentration silver diamine fluoride solution and a 5% sodium fluoride varnish, or (2) the application of glass ionomer cement sealants, atraumatic restorations, and a 5% sodium fluoride varnish. Parental informed consent and child assent at time of treatment were required before children could participate in CariedAway.^17^ The CariedAway clinical research study did not collect any data on dental and dental-related medical care utilization outside of CariedAway. Complete details regarding the study population, informed consent, staffing, and protocols have been previously published.^13^

### Data Sources and Sample Restrictions

We performed a novel linkage between fall 2019 student-level participation data for students who accepted and received treatment through the CariedAway clinical trial to 2016 to 2019 NY Medicaid claims and enrollment data for children residing in NYC using direct identifiers (name, date of birth, gender, and NY Medicaid ID if available) from CariedAway participants. The analytic dataset excluded direct identifiers after linkage. We identified 2632 CariedAway participants in 2019 residing in the Bronx between ages 5 to 13 years identified as NY Medicaid recipients, representing approximately 60% of 2019 CariedAway participants aged 5 to 13 years. This matched the proportion of NYC elementary school children who were enrolled in NY Medicaid in the schools targeted.^18^

We identified elementary school zone assignments for all NY Medicaid-enrolled children (aged 5-13 years) using 2018 to 2019 NYC Open Data. This was based on the beneficiary’s self-reported address at age 5 years, or the earliest address available from 2016 to 2019. This approach accounts for NYC enrollment policies where, at age 5 years, NYC children are eligible to enroll in kindergarten, and are allowed to remain in the same school regardless of subsequent changes in address. The majority of kindergarteners in NYC attend the school they are assigned on the basis of residential address.^19^ We then identified whether children were within school zones with a CariedAway site present within at least 1 of its schools. To ensure comparability between groups, the sample was restricted to the Bronx, where the majority of schools in the CariedAway clinical research study (36 or 46 schools) were located. Finally, we restricted the sample to individuals continuously enrolled in Medicaid from 2018 to 2019 to track their pre-CariedAway utilization. Sample attrition details are summarized in the Figure.

**Figure.**
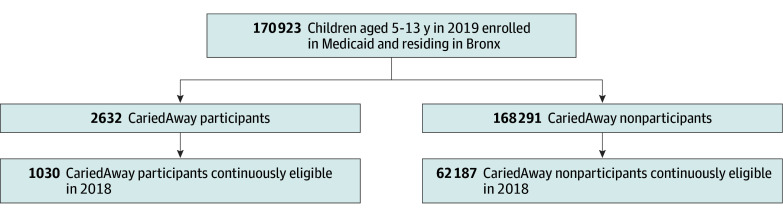
Patient Enrollment Flowchart

Our final analytic sample included CariedAway participants and nonparticipants who had never participated in CariedAway from 2019 to 2023 to avoid comparisons between participants and future participants. For robustness tests, we further restricted the sample to individuals residing in school zones where CariedAway was offered in at least 1 elementary school.

### Medicaid Variables

Using the NY Medicaid claims, we identified nonemergency dental visits to dentists. Dental visits were categorized as nonemergency if they excluded *Current Dental Terminology (CDT)* codes D9110^20^ and D0140.^21^ Nonemergency dental visits were then further categorized as preventive (*CDT* codes D1000-D1999),^22,23^ restorative (*CDT* codes D2000-D2999),^22,23^ endodontic (*CDT* codes D3000-D3999),^22,23^ periodontic (*CDT* codes D4000-D4999),^22,23^ implants and prosthodontics (*CDT* codes D5000-D6999),^22,23^ oral and maxillofacial surgery (*CDT* codes D7000-D7999),^22,23^ and orthodontics (*CDT* codes D8000-D8999).^22,23^ For area-level factors, we included neighborhood (school zone) identifiers to capture time-invariant differences in the health system environment impacting participation in SCPPs. We defined prior experiences with dental care as having any nonemergency dental office visits before the implementation of CariedAway.

Although we cannot directly observe unmet dental need in NY Medicaid claims data, we estimated a proxy using utilization of emergency dental services delivered through either hospital EDs for nontraumatic dental conditions or by dentists. Dental-related medical use in EDs was identified using a set of *International Statistical Classification of Diseases and Related Health Problems, Tenth Revision* codes for nontraumatic dental conditions, according to latest recommendations from state dental directors^7^ and with the most recent addition of the K08/89 *International Statistical Classification of Diseases and Related Health Problems, Tenth Revision* code.^7,8^ Emergency dental visits were captured through *CDT* codes D9110^20^ and D0140.^21^

The Medicaid enrollment file included self-reported race and ethnicity with the following categories: Asian, Hispanic, non-Hispanic Black, non-Hispanic White, other (American Indian or Alaska Native, Native Hawaiian, or Other Pacific Islander), and unknown. Data on race and ethnicity are included here to mitigate omitted variable bias. Because study outcomes are assessed through passive surveillance of NY Medicaid claims data, there was no loss to follow-up or missing values for primary health care utilization outcomes.

### Estimated Impact on Dental-Related ED Charges from a Statewide Rollout

We quantified the potential selection bias of SCPP participation by estimating the percentage point difference due to selection into participation on the basis of prior dental care use and having unmet dental needs using the adjusted probabilities estimated from the logistic regression. We then applied these probabilities to the estimated size of the pediatric Medicaid population with no prior dental care use and with unmet dental needs to calculate the increase in SCPP participation from removing the impact of selection on participation. We finally estimated the averted number of dental-related ED visits among children from treating children who previously selected out of participation and had unmet dental needs.

### Statistical Analysis

First, we characterized the difference in sociodemographic characteristics and dental and dental-related care utilization in 2018, the calendar year preceding CariedAway implementation, among participating and nonparticipating beneficiaries enrolled in NY Medicaid using 2-sample χ^2^ tests for categorical variables (ie, gender and ethnicity) and *t* tests for continuous variables. Statistical significance was assessed at the *P* < .10, *P* < .05, and *P* < .01 levels. To protect participant confidentiality, we excluded cells with fewer than 10 observations.

Second, we used an adjusted logistic regression to assess whether prior dental care use is independently associated with participation after accounting for demographic factors (race and gender), individual-level clinical dental need, and health care system factors, all of which may lead to confounding due to imbalance between participants and nonparticipants. Individual-level clinical dental need was represented by a single binary variable pooling any nontraumatic dental condition ED visits or any dental office emergency visits before CariedAway implementation to improve model stability, since each event is infrequent in both treatment and control groups. A separate regression model estimated the association between CariedAway participation and prior nonemergency dental care use by category (eg, preventive or restorative). School zone–level clustered SEs accounted for correlations among children within school zones.

We removed individuals residing in school zones without CariedAway in schools from the statistical analyses in robustness tests. Because CariedAway systematically chose community locations with demographic characteristics associated with high caries risk, including beneficiaries residing in school zones without CariedAway could overstate potential bias induced by selection into participation.

Data cleaning and processing were conducted in SAS statistical software version 9.4 (SAS Institute). Analysis was conducted using Stata statistical software version 16.1 (StataCorp) after the conclusion of CariedAway in 2023 between June 2023 and October 2025.

## Results

A total of 63 217 children who were continuously enrolled in Medicaid and identified as CariedAway participants (1030 children) or nonparticipants (62 187 children) were included. The children had a mean age of 7.7 years (95% CI, 7.6-7.7 years). There were 30 590 female children (48.4%), 1852 Asian children (2.9%), 13 926 Black children (22.0%), 31 620 Hispanic children (50.0%), and 1988 White children (3.2%). Participants were younger than nonparticipants (mean age, 6.2 years [95% CI, 6.2-6.4 years] vs 7.7 years [95% CI, 7.7-7.7 years]; *P* < .001). Participants were also more likely to be Hispanic and female vs nonparticipants. Both participants and nonparticipants resided in the Bronx in areas without dental professional shortages and with populations that were less than 80% White. Full baseline characteristics comparing participants and nonparticipants are in Table 1.

**Table 1.** Demographic Characteristics of Children

Characteristic	Children, No. (%)			*P* value^a^
	Nonparticipants (n = 62 187)	Participants (n = 1030)	Total (N = 63 217)^b^	
Age, mean (95% CI), y	7.7 (7.7-7.7)	6.3 (6.2-6.4)	7.7 (7.6-7.7)	<.001
Race and ethnicity				
Asian	1828 (2.9)	24 (2.3)	1852 (2.9)	.008
Black	13 725 (22.1)	201 (19.5)	13 926 (22.0)	
Hispanic	31 045 (49.9)	575 (55.8)	31 620 (50.0)	
White	1960 (3.2)	28 (2.7)	1988 (3.2)	
Other^c^	2586 (4.2)	32 (3.1)	2618 (4.1)	
Unknown	11 043 (17.8)	170 (16.5)	11 213 (17.7)	
Sex				
Male	32 142 (51.7)	485 (47.1)	32 627 (51.6)	.003
Female	30 045 (48.3)	545 (52.9)	30 590 (48.4)	

^a^ *P* values were calculated by *t* test for continuous variables and χ^2^ test for binary or categorical variables.

^b^ All of the children resided in areas without dental professional shortages and in areas where the population was less than 80% White.

^c^ Other includes American Indian or Alaska Native, Native Hawaiian, or Other Pacific Islander.

Table 2 shows any differences in dental and dental-related medical care utilization in the calendar year before CariedAway implementation (2018). Participants were more likely than nonparticipants to have had any dental and preventive dental visits, although the difference was not clinically significant as per common Cohen h thresholds. Participants were less likely than nonparticipants to have visited the ED for nontraumatic dental needs, but the difference was not statistically significant.

**Table 2.** Probability of Any Dental Care Utilization in 2018, the Year Before CariedAway Implementation

Variables	Children, No. (%)			*P* value^a^
	Nonparticipants (n = 62 187)	Participants (n = 1030)	All (N = 63 217)	
Any dental office	34 454 (55.4)	606 (58.8)	35 060 (55.5)	.03
Any preventive dental	31 335 (50.4)	553 (53.7)	31 888 (50.4)	.04
Any restorative	8632 (13.9)	147 (14.3)	8779 (13.9)	.72
Any endodontic	1257 (2.0)	23 (2.2)	1280 (2.0)	.63
Any periodontic	23 (<0.1)	Cell size <10	Redacted	.33
Any orthodontic	3070 (4.9)	22 (2.1)	3092 (4.9)	<.001
Any dental emergency department	892 (1.4)	12 (1.2)	904 (1.4)	.47
Any dental office emergency	4009 (6.5)	65 (6.3)	4074 (6.4)	.86

^a^ *P* values were calculated by *t* test for continuous variables and χ^2^ test for binary or categorical variables. We excluded teledental, since there were no claims for teledental services during this period.

Table 3 shows the main logistic regression results, with CariedAway participation as the dependent variable. Having no dental visits was associated with 17% lower odds of participating in CariedAway, all else fixed (adjusted odds ratio [aOR], 0.83; 95% CI, 0.71-0.96). After estimating the association of participation with different types of prior nonemergency dental office utilization, the association seems to be driven primarily by preventive visits, such that having no preventive visits was associated with 14% lower odds of participating in CariedAway, compared with those with preventive visits (aOR, 0.86; 95% CI, 0.74-1.00; *P* < .05). In contrast, there was no statistically significant association between CariedAway participation and no prior restorative visits (aOR, 1.03; 95% CI, 0.79-1.36). Similarly, there was no statistically significant association between having no endodontic, periodontic, or orthodontic visits and CariedAway participation. However, having no emergency dental care utilization was associated with 27% increased odds of participating in CariedAway, compared with those with any emergency dental care utilization across regression specifications (aOR, 1.27; 95% CI, 1.02-1.57). Being female was associated with 24% higher odds of participating in CariedAway compared with the baseline group of male children, across all regression specifications (aOR, 1.24; 95% CI, 1.12-1.37).

**Table 3.** Logistic Regression Examining the Association of Prior Dental Care Utilization in 2018 With CariedAway Participation

Variable	aOR (95% CI)^a^			
	Baseline sample (n = 116 elementary school zones)		CariedAway zones (n = 34 elementary school zones)	
	Model 1 (n = 57 980 observations)	Model 2 (n = 57 980 observations)	Model 3 (n = 18 555 observations)	Model 4 (n = 18 555 observations)
Type of prior nonemergency dental care utilization				
No nonemergency dental office visits	0.83 (0.71-0.96)^b^	NA	0.79 (0.66-0.94)^c^	NA
No preventive visits	NA	0.86 (0.74-1.00)^b^	NA	0.84 (0.71-1.00)^b^
No restorative visits	NA	1.03 (0.79-1.36)	NA	0.92 (0.68-1.25)
No endodontic visits	NA	0.99 (0.71-1.39)	NA	0.89 (0.64-1.25)
No periodontic visits	NA	0.19 (0.02-2.15)	NA	NA
No surgical visits	NA	1.04 (0.75-1.41)	NA	1.21 (0.82-1.78)
No orthodontic visits	NA	0.83 (0.51-1.37)	NA	0.75 (0.38-1.46)
No dental emergency department or dental office emergency visits	1.32 (1.08-1.62)^c^	1.27 (1.02-1.57)^b^	1.27 (1.02-1.58)^b^	1.18 (0.93-1.48)
Female sex	1.24 (1.12-1.37)^c^	1.24 (1.12-1.37)^c^	1.32 (1.16-1.49)^c^	1.32 (1.17-1.49)^c^

Abbreviations: aOR, adjusted odds ratio; NA, not applicable.

^a^ aORs accounted for clustered SEs, with clustering at the school zone level. The category of implants and prosthodontics was excluded from models 2 and 4 because of their rarity in the pediatric Medicaid population. In model 4, the periodontic visit category was excluded because of the rarity of having any periodontic visits in the sample composed of individuals residing in zones with CariedAway. Twenty-nine groups (5242 observations) were excluded from the logistic regression in models 1 and 2, and 4 groups (141 observations) were excluded in models 3 and 4 because all observations in these groups had all positive or all negative outcomes. All models included controls for race and birth year and included elementary school zone identifiers.

^b^ *P* < .05.

^c^ *P* < .01.

The results were largely robust to the choice of analytic sample. After restricting the analyses to children residing in school zones with at least 1 elementary school participating in CariedAway, having no prior dental office visits or preventive visits was still associated with 21% lower odds of participating in CariedAway (aOR, 0.79; 95% CI, 0.66-0.94) (Table 3). In addition, the magnitude of the association between having no emergency dental care utilization and participation remained stable across analytic samples and regression specifications, but with varying statistical significance. The association between being female and CariedAway participation increased in the smaller sample to 32% increased odds of participation compared with male children (aOR, 1.32; 95% CI, 1.16-1.49).

Logistic regression results suggest that selection into participation on the basis of prior dental care use and having unmet dental needs accounted for a 6.2 percentage point difference between groups with the highest and lowest caries risk. Given that 90% of children with unmet dental needs have no prior dental care use, and 2% of pediatric Medicaid beneficiaries have unmet dental needs,^24^ we estimate a total of 37 800 children enrolled in NY Medicaid per year with unmet dental needs. Hence, a rollout of CariedAway to all 2.1 million NY Medicaid enrollees younger than 18 years^25^ would exclude 14 137 children with the highest caries risk. If there were no selection into participation, then an additional 2343 children with unmet dental needs would be enrolled. Since there are at least 16 000 dental ED visits per year within New York among children aged 5 to 13 years in our data, an estimated 42% of children with unmet dental needs visit the ED for dental needs. Using a mean charge of $2337 per dental-related ED visit in 2022,^25^ removing the selection from CariedAway could avert an additional approximately $2.4 million in dental ED visit charges.

## Discussion

In this cross-sectional study, our primary analysis found evidence for selection into SCPPs based on prior dental care use, whereby children with any prior dental care use (primarily preventive) were more likely to select into SCPP participation. Even after limiting the sample to individuals in neighborhoods with SCPPs, the association between prior preventive dental care use and SCPP participation persisted. Hence, SCPP participants were more likely to have had prior preventive dental care use compared with both the Medicaid pediatric population and children residing in the same neighborhoods with similar levels of dental and health care access. This is consistent with previous studies demonstrating prior-year utilization as a factor associated with subsequent health care use.^26,27^

These results suggest that children with nontraumatic dental-related ED or emergency dental visits (proxying unmet dental needs) are opting out of SCPP participation, despite a targeted focus within populations at high caries risk.^16,28^ Additionally, female children are more than 20% more likely to participate in SCPPs, aligning with prior literature on school-based health programs showing girls are overwhelmingly more likely to use school-based health programs.^29,30,31,32,33,34,35,36,37,38,39,40,41,42^

Removing selection bias in a statewide SCPP rollout could save up to $2.4 million in Medicaid dental-related ED visit charges. However, no studies to date have directly examined the long-term downstream impact of SCPPs on dental and dental-related medical care utilization or outcomes in children with high and low caries risk. This lack of data may severely curtail the ability of potential implementors to elicit buy-in from communities, schools, and other stakeholders.

### Limitations

This study has limitations that should be mentioned. Although our sample criteria in the robustness check are aimed at identifying nonparticipants likely to attend schools with CariedAway, the sample may still include children attending out-of-zone schools without CariedAway, private schools, or other schooling arrangements. In addition, limiting the study to continuously insured Medicaid enrollees may overlook those with intermittent eligibility and with less consistent dental care access. Future work should, therefore, seek to more precisely identify nonparticipants who are enrolled in schools with SCPPs but opting out of participation to better understand whether there are flaws in how schools are selected for SCPPs, and to survey them on their prior dental care use and self-assessed dental needs.

Additionally, individuals with increased need may also be those seeking more dental care but still having unmet dental needs. For instance, children may seek dental care and initial evaluations due to unmet dental need but may not follow up with dentists due to substantial costs and barriers to restorative care. In this case, these children may seek care through SCPPs to address continuing unmet dental needs despite having more preventive dental care than nonparticipants. However, at the extreme, such children may also be more likely to seek out emergency dental care either through EDs or dental offices, should their unmet dental need escalate to severe pain or other adverse effects. Future work should collect oral health outcomes and dental-related care utilization data for nonparticipants and participants to fully disentangle prior dental care use from unmet dental need.

Although emergency dental utilization likely includes both caries-related and traumatic dental visits, due to limitations of current *CDT* coding, this biases our results toward the null. Hence, we likely underestimate the impact of unmet dental needs (proxied by emergency dental utilization) on program participation. Future work should directly measure patient-reported levels of unmet dental needs for both participants and nonparticipants.

## Conclusions

In this cross-sectional study introducing a novel linkage between SCPP participation data and NY Medicaid claims data, we found that preventive dental care experience before SCPP implementation was significantly associated with later SCPP participation. Additionally, there was some indication that children at the highest echelons of caries risk did not participate in SCPPs, since having any dental-related emergency care utilization was negatively associated with SCPP participation. We found that reducing the amount of selection into participation may come with substantial savings to Medicaid state programs in the form of decreased spending on ED visits for dental conditions.

## Notes

Supplement. Data Sharing Statement
