# Supplementary material for: School-Based Caries Prevention Programs and Recruitment of High-Risk Pediatric Medicaid Populations
Source: JAMA Netw Open. 2026 Apr 9;9(4):e265996. doi: 10.1001/jamanetworkopen.2026.5996 (PMC13067015; doi:10.1001/jamanetworkopen.2026.5996)
Supplement: Supplement. — Data Sharing Statement [file jamanetwopen-e265996-s001.pdf]

Data Sharing Statement

Huang. School-Based Caries Prevention Programs and Recruitment of High-Risk Pediatric Medicaid Populations. *JAMA Netw Open*. Published April 09, 2026.
doi:10.1001/jamanetworkopen.2026.5996

Data
Data available: No

Additional Information
Explanation for why data not available: Access to the NY Medicaid data requires approval from the New York Department of Health
